# NMR metabolomics of cerebrospinal fluid differentiates inflammatory diseases of the central nervous system

**DOI:** 10.1371/journal.pntd.0007045

**Published:** 2018-12-17

**Authors:** Caitlin D. French, Rodney E. Willoughby, Amy Pan, Susan J. Wong, John F. Foley, L. Joseph Wheat, Josefina Fernandez, Rafael Encarnacion, Joanne M. Ondrush, Naaz Fatteh, Andres Paez, Dan David, Waleed Javaid, Ioana G. Amzuta, Anne M. Neilan, Gregory K. Robbins, Andrew M. Brunner, William T. Hu, Darya O. Mishchuk, Carolyn M. Slupsky

**Affiliations:** 1 Department of Nutrition, University of California, Davis, California, United States of America; 2 Department of Pediatrics, Division of Infectious Disease, Medical College of Wisconsin, Milwaukee, Wisconsin, United States of America; 3 Wadsworth Center Diagnostic Immunology Laboratory, New York State Department of Health, Albany, New York, United States of America; 4 Intermountain Healthcare, Salt Lake City, Utah, United States of America; 5 Department of Medicine, Division of Infectious Diseases, Indiana University School of Medicine, Indianapolis, Indiana, United States of America; 6 Hospital Infantil Robert Reid Cabral, Santo Domingo, Distrito Nacional, República Dominicana; 7 Inova Fairfax Hospital, Fairfax, Virginia, United States of America; 8 Departamento de Ciencias Basicas, Universidad de la Salle, Bogotá, Colombia; 9 Rabies Lab, Kimron Veterinary Institute, Beit Dagan, Israel; 10 Department of Medicine, SUNY Upstate Medical University, Syracuse, New York, United States of America; 11 Department of Medicine, Division of Infectious Diseases, Massachusetts General Hospital, Boston, Massachusetts, United States of America; 12 Mayo Clinic, Rochester, Minnesota, United States of America; 13 Department of Food Science and Technology, University of California, Davis, California, United States of America; Wistar Institute, UNITED STATES

## Abstract

**Background:**

Myriad infectious and noninfectious causes of encephalomyelitis (EM) have similar clinical manifestations, presenting serious challenges to diagnosis and treatment. Metabolomics of cerebrospinal fluid (CSF) was explored as a method of differentiating among neurological diseases causing EM using a single CSF sample.

**Methodology/Principal findings:**

^1^H NMR metabolomics was applied to CSF samples from 27 patients with a laboratory-confirmed disease, including Lyme disease or West Nile Virus meningoencephalitis, multiple sclerosis, rabies, or *Histoplasma* meningitis, and 25 controls. Cluster analyses distinguished samples by infection status and moderately by pathogen, with shared and differentiating metabolite patterns observed among diseases. CART analysis predicted infection status with 100% sensitivity and 93% specificity.

**Conclusions/Significance:**

These preliminary results suggest the potential utility of CSF metabolomics as a rapid screening test to enhance diagnostic accuracies and improve patient outcomes.

## Author summary

Inflammation of the brain and spinal cord, known as encephalomyelitis, is a dangerous condition that can be caused by a wide range of pathogens, such as viruses and bacteria, and other medical conditions including autoimmunity or drug intoxications. Given the many possible causes, it is often difficult for clinicians treating patients with encephalomyelitis to identify the underlying cause, which in turn determines the appropriate treatment. Infections and other diseases causing neurological inflammation work by distinct biological mechanisms and, consequently, can cause unique biochemical changes that can be observed in cerebrospinal fluid of affected individuals. The researchers used a metabolomics technique to measure a range of small molecules in cerebrospinal fluid and examine biochemical differences in patients with encephalomyelitis caused by Lyme disease, West Nile Virus, multiple sclerosis, rabies, or fungal infection. The researchers found distinct differences in the biochemical profiles of patients whose encephalomyelitis was caused by infections versus patients with no infection, and also identified different patterns among the individual diseases. This study showed that metabolomics may be useful in improving diagnosis and treatment of diseases affecting the central nervous system by enhancing understanding of their unique effects on metabolism.

## Introduction

Encephalomyelitis (EM) is a condition characterized by inflammation of the brain (encephalitis) and spinal cord (myelitis) that frequently causes permanent disability. There are myriad causes of EM syndromes, which are in aggregate relatively common [1–4] and include viral, bacterial, fungal, protozoal and prion infections, autoimmune encephalitis, intoxications, and metabolic encephalopathies, while other EM cases have unknown causes [5]. Clinicians face significant challenges to the rapid and accurate diagnosis and treatment of EM. Due to the rarity of a definitive diagnosis, many arbovirus and other viral causes of EM, including rabies, have limited evidence-based therapies; this may change with newer broad-spectrum antivirals currently in clinical trials [6]. Treatment of autoimmune EM relies on corticosteroids, immunoglobulin, plasmapheresis, cytotoxic agents and biologicals [7, 8], which are typically contra-indicated until infections can be excluded. Physicians are often forced to treat empirically for infections and delay appropriate therapy for autoimmune EM, thereby worsening patient outcomes. Moreover, for many causes of EM, no rapid diagnostic testing exists, and long delays pending laboratory test results commonly occur before definitive treatment may be initiated; however, superior outcomes depend on early intervention. Because there are numerous causes of EM, including multiple infectious agents that overlap or coincide in geographic distribution, diagnosis reliant on single-target testing is unsatisfactory as it requires quantities of tests that are not only prohibitive in cost but also involve collecting unsafe volumes of blood or cerebrospinal fluid (CSF) from patients. Improved diagnostics and proxy markers of therapeutic efficacy are sorely needed, especially as new treatment regimens develop.

In recent years, the development and expansion of omics technologies have presented opportunities for discovering disease mechanisms and biomarkers of clinical significance [9–11]. Metabolomics, the comprehensive study of small-molecule metabolites in a biofluid or tissue, offers a set of clues to the biochemical workings of a body system, organ, or compartment in a given physiological state, and has diverse applications in improving clinical diagnosis and treatment of central nervous system (CNS) diseases and intoxications [9, 12–17]. Metabolomics panels may also provide information about a broad spectrum of metabolic processes involved in a disease presentation compared to traditional single-molecule assays. Metabolites present in CSF may originate from brain metabolic processes, including intermediate and end products of energy metabolism, neurotransmission, inflammation and oxidative stress responses; thus, their analysis provides insights into metabolic disturbances occurring in CNS diseases. Among the methodological approaches taken in metabolomics studies of CSF, ^1^H-NMR spectroscopy carries advantages for exploratory studies both in the scope of metabolite detection and its quantitative ability [18]. An additional advantage of this method is the lack of sample consumption, given practical limitations on the volume of CSF usually available. Further, many CNS diseases and intoxications are prevalent in countries where advanced imaging facilities, reference laboratories and therapeutics are in short supply. Recent studies have applied ^1^H NMR-based metabolomics of CSF to identify single-molecule biomarkers and panels of metabolites associated with a range of neurological diseases such as infectious meningitis [14], multiple sclerosis (MS) [13, 19, 20], Alzheimer’s [21, 22] Parkinson’s [23] and Huntington’s diseases [24]. Further, this method has detected metabolic changes characterizing different stages of disease progression in rabies and MS [12, 25]. Proxy markers of disease progression or response to therapy may also accelerate therapeutic trials while lowering their cost.

Despite significant advances in the application of NMR metabolomics in the investigation of certain CNS diseases, such as multiple sclerosis, its potential to describe metabolic changes occurring in many infectious neurological diseases has been less studied. Lyme disease and West Nile Virus (WNV) are vastly under-studied in this sense, despite being the most common causes of vector-borne bacterial and viral disease, respectively, in the United States [26, 27]. Rabies is an important global zoonosis but may be underdiagnosed in some contexts due to challenges in distinguishing it clinically from other CNS infections, such as cerebral malaria, in areas where these are endemic [28]. Infectious diseases that invade the CNS have distinct molecular mechanisms driving their respective pathologies [29, 30]. Further, pathogen strategies to replicate while evading host immune responses can involve the disruption of a range of endogenous metabolic processes [31], many of which have yet to be illuminated for specific diseases; thus, explorative studies of the CSF metabolome in different disease states can provide an important window for examining potential pathogen effects on metabolism within the CNS to lay the groundwork for future targeted diagnostics or therapeutic interventions. In the present study CSF samples from patients representing diverse infectious and non-infectious diseases of the CNS were analyzed by ^1^H NMR-spectroscopy to determine if metabolomics profiling could distinguish diseases. We find preliminary evidence of the existence of discriminating metabolic features.

## Methods

### Subjects

Twenty-seven patients were diagnosed with CNS Lyme disease (n = 5, all ages, at the New York State Department of Health), WNV meningoencephalitis (n = 5, all ages, New York State Department of Health), Clinically Isolated Syndrome (CIS) of multiple sclerosis (MS, n = 4, adults, Intermountain Healthcare), rabies (n = 10, all ages, at Canadian Food Inspection Agency, Centers for Disease Control and Prevention, Kimron Veterinary Institute, National Institutes of Health-Colombia, and New York State Department of Health), or *Histoplasma* meningitis (n = 3, anonymous, at Indiana University School of Medicine). Due to ethical concerns surrounding the collection of CSF from healthy individuals, healthy controls were not available for this study. Specimens obtained as discard material from 25 anonymous children aged 5–20 years at the Children’s Hospital of Wisconsin with no concurrent microbiological testing and no known encephalopathy or encephalitis served as a control group. This population includes mostly children with cancer in remission or children being treated for pseudotumor cerebri, a common non-inflammatory condition. Given patient samples were anonymous discard material, the study was ruled to not be human research requiring informed consent by the Children’s Hospital of Wisconsin IRB (protocol CHW 10/24). For rabies patients, for whom multiple specimens were available, the specimen taken closest to the fourth day of hospital admission was selected to minimize the influence of hypoglycemia, ketosis or renal insufficiency on presentation to the CSF metabolome. While the CSF was collected for diagnostic purposes, precise timing is uncertain other than for rabies patients. Initially, four specimens from patients with *Histoplasma* meningitis were analyzed, but one specimen had a metabolite profile inconsistent with CSF and was excluded on the basis of containing implausible values. Three *Histoplasma* specimens remained after this exclusion.

### Storage and preparation of CSF samples

After collection, specimens were stored refrigerated and/or frozen until transport on dry ice to the site of analysis, where they were stored at -80°C until sample preparation. Once defrosted, samples were filtered using washed Amicon Ultra-0.5 mL centrifugal filters with a cut-off of 3000 MW (Millipore, Billerica, MA) to remove lipids and proteins. When needed, filtrate volume was adjusted to 207 μL when preparing for 3mm NMR tubes or 585 μL when preparing for 5mm NMR tubes with Type I ultrapure water from Millipore Synergy UV system (Millipore, Billerica, MI). Samples were prepared for analysis by the addition of 23 μL or 65 μL of internal standard containing approximately 5 mmol/L of DSS-d6 [3-(trimethylsilyl)-1-propanesulfonic acid-d6], 0.2% NaN_3_, in 99.8% D_2_O to 207 μL or 585 μL of CSF filtrate, respectively. The pH of each sample was adjusted to 6.8 ± 0.1 by adding small amounts of NaOH or HCl. A 180 or 600 μL aliquot was subsequently transferred to 3 mm or 5mm Bruker NMR tubes, respectively, and stored at 4 ^o^C until NMR acquisition (within 24 hours of sample preparation). NMR spectra were acquired as previously described [12] on a Bruker Avance 600-MHz NMR equipped with a SampleJet autosampler using a NOESY-presaturation pulse sequence (noesypr) at 25°C.

### Data analysis

NMR spectra were manually phased and baseline-corrected using NMR Suite v6.1 Processor (Chenomx Inc., Edmonton, Canada), and Chenomx NMR Suite v.8.1 Profiler (Chenomx Inc., Edmonton, Canada) was used for quantification of metabolites. Selected NMR spectral data from a previous rabies study in this lab [12] were compared to additional samples acquired from Lyme, WNV, histoplasmosis and MS patients.

After correcting metabolite concentrations for dilution, data were cluster-analyzed 2 ways for comparison using RStudio software (RStudio Version 1.0.136, Boston, MA, USA) or Stata software (SE 14, College Station, TX, USA). First taking a data-driven approach, concentrations were log_10_-transformed before principal component analysis (PCA) was carried out on the covariance matrix of the centered data as an unsupervised search for trends. Alternatively, to provide clinical context, data were normalized to z-scores using published reference ranges in CSF (www.hmdb.ca). In instances when published norms were discrepant, those that encompassed the range of our control population were selected. In rare instances when normal ranges were unavailable, means and standard deviations were constructed using our 25 controls. Normalization by z-scores constructed from population norms generated more skewed data than log_10_-transformation across the entire spectrum of diseases and controls. Factor analysis better tolerates skewed data than PCA and was applied to the z-scores.

Based on the separation found by PCA and factor analysis, differences in metabolite concentrations by infection status and by individual disease diagnoses were assessed on the untransformed data using Mann-Whitney *U* tests and Kruskal-Wallis tests, respectively. P-values were adjusted for multiple comparisons using false discovery rates. Homogeneity of variance between groups was tested using the Levene test to inform interpretation of the rank sum test results. For metabolites with significant differences by Kruskal-Wallis testing, Dunn’s multiple comparisons tests were performed between each pair of groups to determine which diseases were different from each other. For these tests, p-values were Bonferroni-adjusted within the 15 multiple comparisons carried out for each metabolite. After adjustment, p-values of less than 0.05 were considered significant. Cliff’s Delta statistics [32] were calculated to assess the degree of overlap in metabolite concentrations by infection status and between diseases that were found to have significant differences by the Dunn’s test.

Untransformed data were also analyzed by predictive analysis [33, 34]. Classification and regression trees (CART) and Random Forests were performed using Salford Predictive Modeler software suite CART and suite Random Forests (Salford Systems, San Diego, CA, USA). For CART, parent node and terminal node were 10 and 5, respectively. 10% leave-out samples were used for cross-validation. Random Forests are collections of decision trees, and each tree was grown on a random (~2/3) subsample of the data. The remaining data were used to determine the performance of the trees. The number of trees to build was 1000. The number of predictors considered for each node was the square root of the number of potential predictors, and the parent node minimum cases was 2. The variable importance was assessed using the GINI method. Target variable and predictors were the same as for CART.

## Results

CSF samples obtained from 25 controls and 27 patients with different neurological diseases were analyzed by 1H-NMR spectroscopy. Table 1 summarizes clinical characteristics of patients included in this study. A total of 57 compounds were identified and quantified in CSF samples; rabies spectra from a prior study [12] were repeat-profiled. Quantification for 13 metabolites present at very low concentrations in a majority of samples was considered not to be exact (S1 and S2 Tables) but still useful in detecting differences between groups. To further minimize the reversible behavioral effects of starvation and dehydration in the analysis normalizing by z-scores, we excluded 3 ketone bodies (3-hydroxybutyrate, acetoacetate, and acetone) and creatinine from the dataset.

**Table 1:** Patient characteristics.

Diagnosis (*n*)	Age, median (range)	Sex, female count (%)	Location
**Rabies (10)**	8 (4–63)	2 (20)	Global^1^
**West Nile Virus (5)**	65 (53–83)	2 (40)	USA
**Lyme Disease (5)**	19 (7–55)	1 (20)	USA
**Fungal (3)**	unknown	unknown	USA
**Multiple Sclerosis (4)**	31 (22–49)	3 (75)	USA

^1^Samples came from USA, Colombia, Dominican Republic, and Equatorial Guinea

### Metabolite profiles by infection status

**Fig 1:**
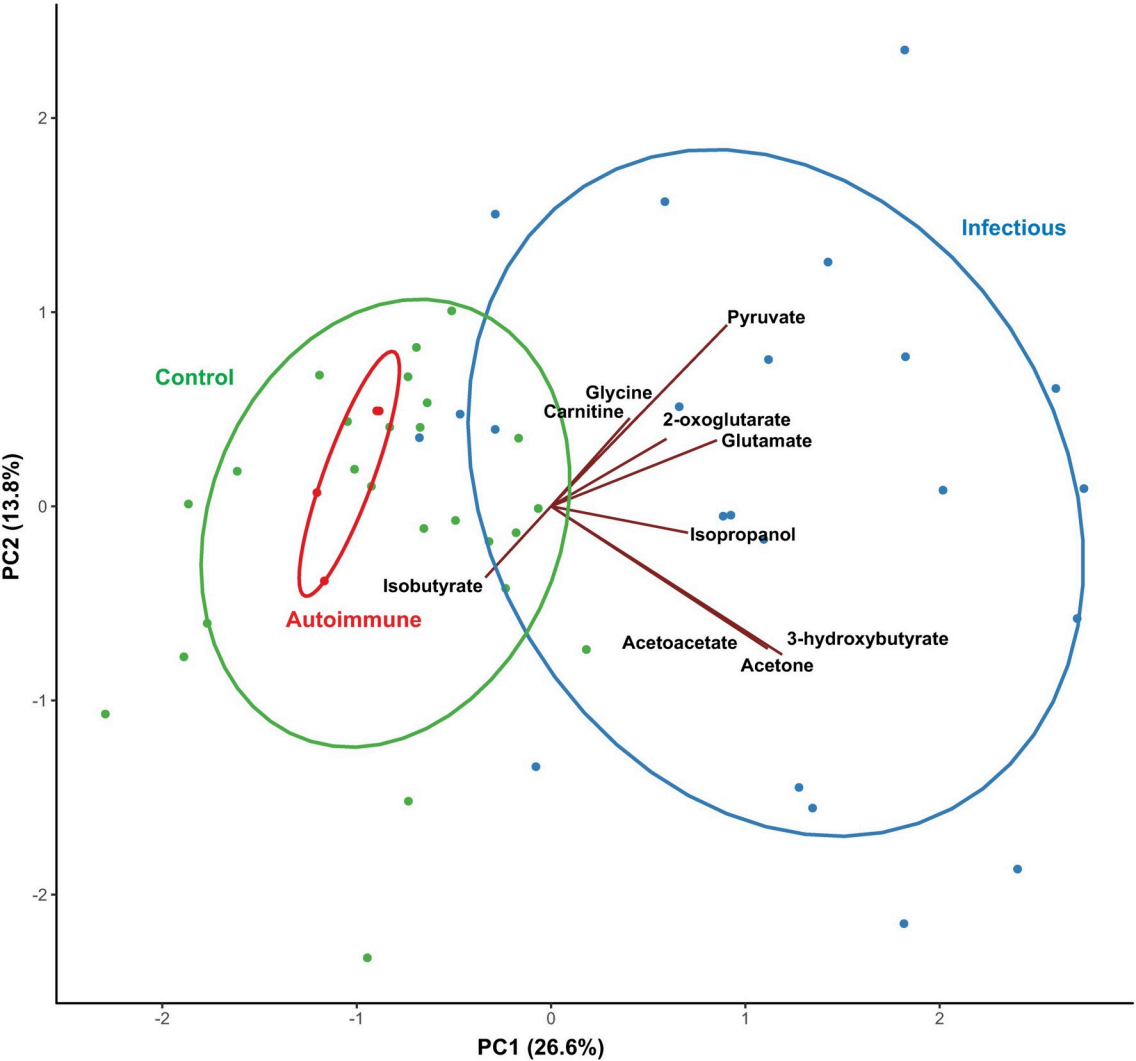
Principal component analysis of ^1^H-NMR CSF metabolomic data comparing infectious or autoimmune disease or controls. Axes represent principal component (PC) scores. The percent of the variation explained by each component is given in parentheses. Green circles represent control patient samples, red circles represent multiple sclerosis patient samples, and blue circles represent infectious disease patient samples. Normal data ellipses are shown for each group. Vectors represent loadings of select metabolites with PC1 and PC2, as drawn in the Gabriel’s biplot. Groups of vectors that point in similar directions tend to change together. One extreme observation was excluded to better visualize the data.

A major clinical challenge is determining whether infection exists as a contraindication to immunosuppression. Unsupervised PCA was performed on metabolite data from patients diagnosed with a neurological disease and controls. Six compounds (acetaminophen, ethanol, ethylene glycol, glycerol, propylene glycol, and valproate) of likely exogenous origin were excluded from cluster analysis models. The first two principal components (PC) in this model accounted for 37.8 percent of the variation in metabolite concentrations. Prominent overlap was apparent between controls and MS, which separated distinctly from infectious diseases along PC 1 (Fig 1). In a scores plot of the first two components, PC 2 identified an apparent outlier in the WNV group, which upon closer examination was observed to have extremely low levels of citrate, lactate, and amino acids coupled with markedly high glutamate, pyruvate, acetate and 2-oxoglutarate compared to the rest of the samples. Since the general patterns generated by PCA did not change when this individual was removed from the dataset, the results shown in Fig 1 reflect this exclusion in order to better visualize clusters in the data.

When overlaid with loadings vectors, the scores plot of the first two PCs revealed two patterns of metabolites among infectious diseases, one characterized by higher levels of ketone bodies and the other by higher levels of pyruvate, glutamate, 2-oxoglutarate, carnitine, and glycine (Fig 1). Pearson correlation coefficients reflect moderate to high correlation among the metabolites in each pattern, with correlation coefficients ranging from 0.77 to 0.93 among ketone bodies and from 0.23 to 0.58 among metabolites in the second pattern. In contrast, metabolites including acetate, isobutyrate, myo-inositol, threonine, and glutamine appeared to characterize controls and MS using loadings vectors.

The contribution of ketone bodies to the PCA analysis prompted a second, clinically applicable analysis using z-scores of normal human values for each metabolite while excluding the potentially non-specific markers of dehydration and starvation, which yielded similar results. Unsupervised factor analysis discriminated CNS disease from controls, with 2 factors accounting for 35.6 percent of the variation. The WNV sample that appeared as an outlier by PCA was not influential in this analysis. Factor analysis excluding ketones and creatinine did not discriminate infections from normal as well as did the PCA analysis.

Given the graphical separation by infection status shown by PCA and factor analysis, Mann-Whitney *U* tests were performed to test for differences in metabolite concentrations between patients with an infectious CNS disease and those with no CNS infection (MS and controls). All metabolites were included. These results are summarized in Table 2. After correcting for multiple comparisons, significant univariate differences were detected in the concentrations of 29 compounds; these included several metabolites that appeared to drive separation in the PCA (ketones, pyruvate, carnitine, and glycine). Median concentrations of glutamate and 2-oxoglutarate were significantly higher in infectious diseases than patients with no infectious disease, and there was a trend towards higher citrate concentrations in the infectious disease group (p = 0.07). Also, in agreement with the PCA results, median concentrations of isobutyrate, fructose, N-acetylneuraminate, and serine were higher in the noninfectious disease group, and acetate exhibited different distributions between the groups. In a similar univariate analysis on z-scores for 43 variables, nine metabolites were identified (Table 2, among bolded metabolites), all of which were also identified using the previous method.

**Table 2:** Differences in cerebrospinal fluid metabolite concentrations between patients with an infectious disease (*Hisotoplasma*, Lyme, rabies, West Nile virus) and non-infectious conditions (multiple sclerosis, controls)^a^.

Metabolite	Potential Pathway(s) Involved	Effect Size^b^	P-value^c^
**Betaine**	1-carbon metabolism/ cell volume/choline oxidation	0.58	0.0022
**Formate^d^**	1-carbon metabolism/acetate synthesis	0.68	<0.0001
**Glycine^e^**	1-carbon metabolism/glutathione synthesis/excitotoxicity	0.62	0.0011
**Choline^e^**	1-carbon metabolism/lipid turnover	0.45	0.0166
**Serine**	1-carbon metabolism/protein catabolism	-0.52	0.0059
**Pyroglutamate^e^**	Amino acid/glutathione metabolism	0.80	<0.0001
***N-*Acetylneuraminate**	Amino sugar metabolism/innate immunity	-0.48	0.0117
**Fructose**	Carbohydrate	-0.52	0.0059
**Acetate^e^**	Carbohydrate and fatty acid metabolism	-0.44	0.0182
**2-Oxoglutarate**	Cell volume/TCA cycle/amino acid metabolism	0.39	0.0387
***N-*Acetylaspartate**	Cell volume/TCA cycle/myelin synthesis	0.37	0.0499
**Glutamate**	Cell volume/TCA cycle/neurotransmitter	0.82	<0.0001
**Methanol^f^**	Drug metabolite; microbial metabolism	-0.46	0.0140
**Pyruvate^d^**	Energy metabolism	0.55	0.0007
**Lactate^e^**	Energy metabolism	0.21	0.0059
**Carnitine^d^**	Fatty acid catabolism	0.54	0.0009
**2-Hydroxybutyrate^e^**	Glutathione synthesis/energy metabolism	0.65	0.0007
**Acetone^e^**	Ketone bodies	0.70	0.0002
**3-Hydroxybutyrate^e^**	Ketone bodies	0.59	0.0022
**Acetoacetate^e^**	Ketone bodies	0.51	0.0070
**Isobutyrate**	Microbial metabolism	-0.41	0.0292
**Isopropanol**	Microbial/ketone metabolism	0.76	0.0001
**2-Oxoisocaproate^d^**	Organic acid	0.39	0.0170
**Urea**	Protein catabolism	0.38	0.0444
**Phenylalanine**	Protein catabolism	0.37	0.0494
**Quinolinate^d^**	Tryptophan metabolism/excitotoxicity	0.51	0.0016
**Ornithine^d^**	Urea cycle	0.36	0.0258
**3-Hydroxyisobutyrate^d^**	Valine metabolism	0.40	0.0150

^a^Metabolites identified by factor analysis using z-score normalization are shown in bold. ^b^Cliff’s Delta d statistic. Positive values indicate increased concentrations in patients with an infection compared to no infectious disease.

^c^Result of Mann-Whitney *U* test.

^d^Difference in median concentrations confirmed after log transformation of the data improved homoscedasticity. P-value reported corresponds to test performed on log-transformed data.

^e^Assumption of equal variances not met. P-value reported corresponds to test performed on log-transformed data.

^f^Tentatively assigned.

### Metabolite profiles by CNS disease

**Fig 2:**
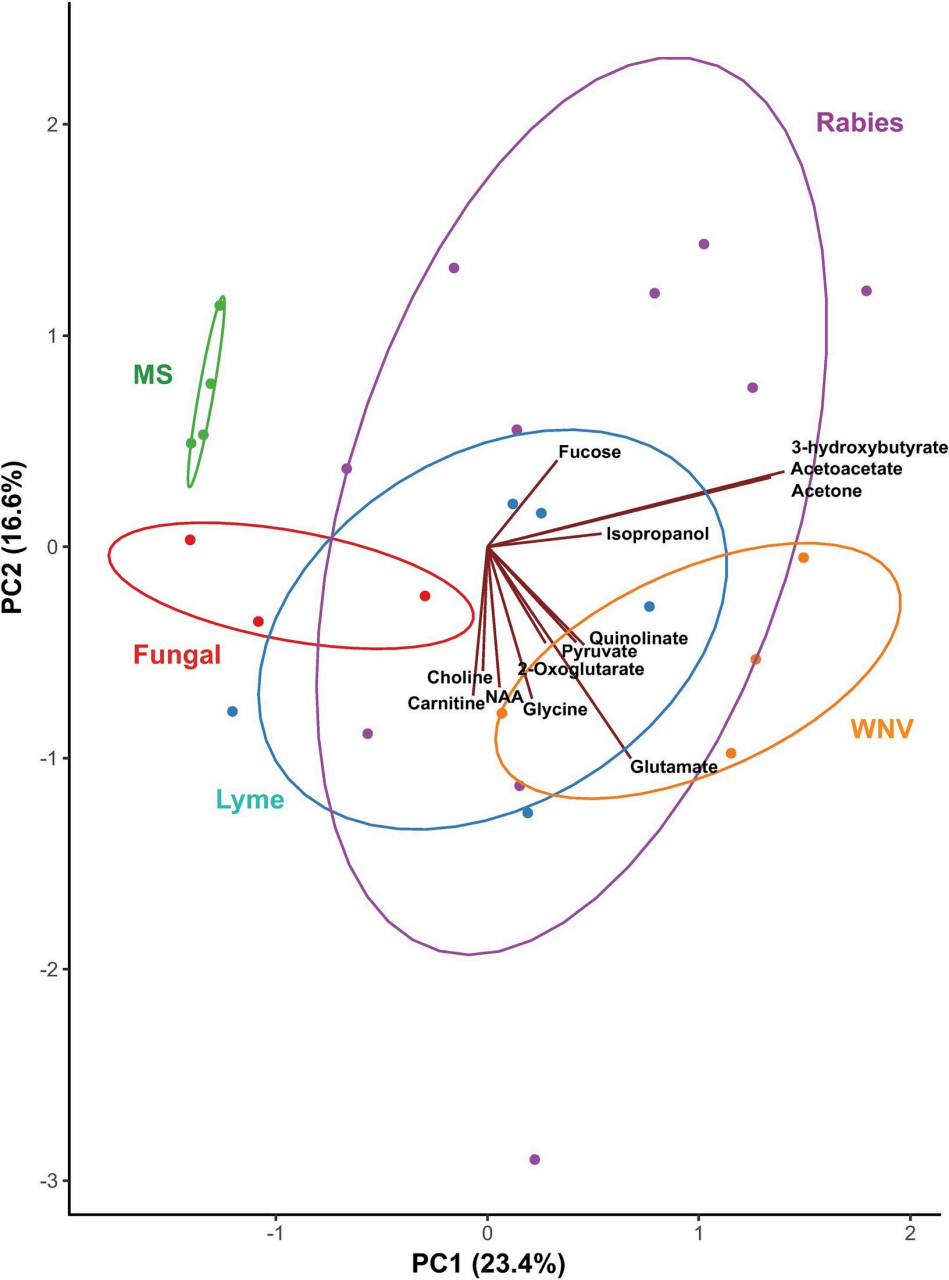
Principal component analysis of ^1^H-NMR CSF metabolomic data comparing neurological diseases. Samples were collected from patients with multiple sclerosis (MS, represented in green), fungal infection (red), Lyme disease (blue), West Nile virus (WNV, orange), and rabies (purple). Normal data ellipses are shown for each group. Axes represent principal component (PC) scores. The percent of the variation explained by each component is given in parentheses. Vectors represent loadings of select metabolites with PC1 and PC2, as drawn in the Gabriel’s biplot. Groups of vectors that point in similar directions tend to change together. One extreme observation was excluded.

While CNS infections overlap as a syndrome, they are caused by viruses, bacteria, fungi, protozoa and prions that require different therapies. We therefore evaluated PCA discrimination within CNS diseases without the influence of controls. In the resulting model, PC1 and PC2 cumulatively accounted for 38.9 percent of the variation, and when loadings vectors were overlaid with PC scores, the resulting Gabriel’s biplot revealed the most important metabolites to be ketone bodies, glutamine, glutamate, and threonine. In a scores plot of the first two PCs, moderate separation by disease diagnosis pointed to differential as well as overlapping metabolic patterns among diseases (Fig 2), which were further dissected in additional analyses and are summarized in Tables 3 and 4. After removing ketones and creatinine, factor analysis of z-scores did not separate cleanly between disease groups.

**Table 3:** Metabolites with differences in cerebrospinal fluid concentrations between two or more disease groups^a^.

Metabolite	Potential Pathway(s) Involved	Adjusted P-value
**Formate^b^**	1-carbon metabolism/acetate synthesis	0.0003
**Glycine^c^**	1-carbon metabolism/glutathione synthesis/excitotoxicity	0.005
**Choline**	1-carbon metabolism/lipid turnover	0.043
**Serine**	1-carbon metabolism/protein catabolism	0.038
**Glutamine^c^**	Amino acid metabolism	0.023
**Pyroglutamate^b^**	Amino acid/glutathione metabolism	0.0003
***N*-Acetylneuraminate**	Amino sugar metabolism/innate immunity	0.013
**Betaine**	Cell volume/1-carbon metabolism/choline oxidation	0.008
**NAAG**	Cell volume/neurotransmitter	0.021
**2-Oxoglutarate**	Cell volume/TCA cycle/amino acid metabolism	0.030
**Glutamate**	Cell volume/TCA cycle/neurotransmitter	0.0003
**Methanol^d^**	Drug metabolite; microbial metabolism	0.043
**Glucose^b^**	Energy metabolism	0.002
**Lactate^c^**	Energy metabolism	0.039
**Pyruvate^b^**	Energy metabolism	0.003
**Carnitine**	Fatty acid catabolism	0.002
**2-Hydroxybutyrate^b^**	Glutathione synthesis/energy metabolism	0.002
**3-Hydroxybutyrate^b^**	Ketone bodies	0.031
**Acetoacetate**	Ketone bodies	0.009
**Acetone^c^**	Ketone bodies	0.002
**Dimethyl sulfone**	Microbial metabolism	0.011
**Isopropanol**	Microbial/ketone metabolism	0.002
**2-Hydroxyisovalerate**	Organic acid	0.018
**Phenylalanine**	Protein catabolism	0.033
**Quinolinate**	Tryptophan metabolism/excitotoxicity	0.018
**3-Hydroxyisobutyrate**	Valine metabolism	0.010
**Acetamide**		0.002

Abbreviation: NAAG, N-Acetylaspartylglutamic acid. ^a^Kruskal-Wallis test for nonparametric one-way analysis of variance, adjusted for multiple tests using the false discovery rate correction.

^b^Difference in median concentrations confirmed after log transformation of the data improved homoscedasticity. ^c^Assumption of equal variances not met.

^d^Tentatively assigned.

**Table 4:** Differences in cerebrospinal fluid metabolite concentrations compared to controls, by disease.

		Cliff’s Delta Statistic^a^			
Metabolite	Potential Pathway(s)	Rabies	West Nile Virus	Lyme Disease	Fungal
**3-hydroxybutyrate^b^**	Ketone bodies	0.64*			
**3-hydroxyisobutyrate**	Valine metabolism	0.62*			
**Acetoacetate**	Ketone bodies	0.75*			
**Lactate^c^**	Energy metabolism	0.62*			
**Isopropanol**	Microbial/ketone metabolism	0.83**^d^		0.89**	
**Glutamate**	Cell volume/TCA cycle/neurotransmitter	0.67^d^	0.96*^d^	0.83*	
**2-hydroxybutyrate^b^**	Glutathione synthesis/energy metabolism	0.89**	0.87**		
**Acetone^c^**	Ketone bodies	0.61*	0.92**		
***N*-Acetylneuraminate**	Amino sugar metabolism/innate immunity		-0.93**		
**Pyruvate^b^**	Energy metabolism		1.00***		
**Betaine**	Cell volume/1-carbon metabolism/choline oxidation		1.00**		
**2-hydroxyisovalerate**	Organic acid		0.81*		
**Carnitine**	Fatty acid catabolism		0.98**		0.95*
**Pyroglutamate^b^**	Amino acid/glutathione metabolism		1.00**	1.00**	1.00*
**Glycine^c^**	1-carbon metabolism/glutathione synthesis/excitotoxicity		0.98**	0.97*	
**Quinolinate**	Tryptophan metabolism/excitotoxicity		0.90*	0.97*	
**Formate^b^**	1-carbon metabolism/acetate synthesis		0.98***	0.98**	
**Glutamine^c^**	Amino acid metabolism			-1.00*	

^a^Estimate of effect size as degree of non-overlap in concentration distributions, where 0 indicates complete overlap and 1 or -1 indicates complete non-overlap. Significance level is indicated by Dunn’s multiple comparisons test. ^b^Difference confirmed after log transformation of the data improved homoscedasticity. Significance level reflects test result on log-transformed data. ^c^Assumption of equal variances not met. ^d^Result when one extreme value was removed as a conservative measure, which attenuated the effect size and p-value. *P<0.05

**P<0.01

***P<0.001

After correcting for multiple comparisons, Kruskal-Wallis tests on untransformed data detected significant differences among diseases and controls in the concentrations of 31 metabolites. Metabolites and diseases for which concentrations were significantly different from control samples according to Dunn’s multiple comparisons tests are shown in Table 4. In particular, the CSF of WNV patients had markedly higher concentrations of pyruvate (p = 0.0008) and formate (p = 0.0005), and Lyme disease and WNV patients shared higher levels of formate and glycine compared to controls. Rabies patients had significantly different concentrations of energy-related metabolites including ketone bodies, lactate and 2-hydroxybutyrate, some of which were also elevated in WNV but not in histoplasmosis or Lyme disease.

### Predictive analyses by infection status and CNS disease

CART analysis differentiated infection status with 100% sensitivity and 93% specificity (Table 5). High pyroglutamate alone discriminated WNV, Lyme and histoplasmosis from controls. MS or rabies could be identified from controls with 100% sensitivity and 76% specificity by high 2-hydroxybutyrate or low 2-hydroxybutyrate and high carnitine. Random Forest analyses confirmed the importance of the majority of metabolites identified by CART.

**Table 5:** Predictive analyses using classification and regression trees (CART) and random forest importance scores.

Prediction	Predictor(s)	Sensitivity	Specificity	Random Forest Relative Importance Score^a^
**Encephalomyelitis vs controls**	Pyroglutamate >35.44 μM or Pyroglutamate ≤35.44 μM & NAAG <1.04 μM	**96.3%**	**96%**	Pyroglutamate 99.94 NAAG 46.65
**Infection vs not (MS + controls)**	Pyroglutamate >35.44 μM or Pyroglutamate ≤35.44 μM & Glucose > 3.92 mM	**100%**	**93%**	Pyroglutamate 100 Glucose 7.96
**WNV, Lyme, histoplasmosis vs controls**	Pyroglutamate >35.67 μM	**100%**	**100%**	Pyroglutamate 100.00
**Rabies, MS vs controls**	2-hydroxybutyrate > 41.61 μM or 2-hydroxybutyrate ≤41.61 μM & Carnitine >2.15 μM	**100%**	**76%**	2-hydroxybutyrate 96.65 Carnitine 20.33
**WNV, Lyme, histoplasmosis vs rabies, MS**	Acetamide > 1.98 μM Betaine > 3.33 μM	**84.6%**	**100%**	Acetamide 100 Betaine 34.53

Abbreviations: NAAG, N-Acetylaspartylglutamic acid; MS, Multiple Sclerosis; WNV, West Nile Virus

^a^GINI method was used, 100 representing the highest score

## Discussion

NMR metabolomics distinguished infectious and inflammatory disorders using laboratory-confirmed samples of 5 disorders using 2 approaches to normalization of the data, and 2 unsupervised cluster analytical approaches. CART decision analysis easily differentiated bacterial (Lyme), fungal (*Histoplasma*) and viral (WNV) causes of encephalomyelitis from controls. Decision analysis also differentiated rabies and the prodromal form of MS from controls, while separation by cluster analyses was incomplete between MS and controls. Notably, the greatest source of variation in metabolomics data found by PCA was the presence or absence of an infectious pathogen. If replicated, this finding is of paramount clinical impact because treatments for infections require almost polar opposite therapeutics than those for autoimmune diseases. There was also substantial agreement in the identification of influential metabolites between different approaches to data normalization and reduction and predictive approaches, including CART and random forest analysis. Metabolites driving separation in PCA (pyruvate, glutamate, quinolinate, 2-oxoglutarate, carnitine, and glycine) potentially suggest alterations in energy metabolism, excitotoxicity and antioxidant response. Patterns of these metabolites were not uniform. Rather, overlapping as well as distinguishing metabolic features were seen, highlighting the potential utility of measuring a suite of metabolites rather than searching for individual metabolic biomarkers for diseases, which may not exist. Overlap of profiles makes strong clinical sense given that EM syndromes overlap in signs and symptoms. The overlap also supports a clinical rationale for syndromic metabolic therapies across a range of infectious or autoimmune causes of EM. Distinguishing features provide promise of rapid, relatively specific diagnoses that enable prompt pathogen or process-directed therapies.

Significant differences by disease group were found in the CSF concentrations of several metabolites known to be involved in the synthesis of the antioxidant glutathione (GSH) and related pathways, including glycine, formate, pyroglutamate, and 2-hydroxybutyrate. The transsulfuration pathway links the methylation cycle of one carbon metabolism to GSH synthesis and produces 2-hydroxybutyrate as a secondary byproduct during the conversion of cystathionine to cysteine [35, 36]. Formate, an endogenous and bacterial metabolite that along with glycine was found at significantly higher levels in WNV and Lyme disease patients compared to controls in this study, is formed as a byproduct in several pathways including the tryptophan kynurenine pathway [37], pterin metabolism [38] and protein demethylation (following hypermethylation by S-adenosyl-L-methionine [39]), while it is also consumed in the folate cycle during the conversion of tetrahydrofolate (THF) to 10-formyl-THF [40]. An end product of purine catabolism, neopterin, has been found to be elevated in patients with rabies [41], Lyme disease, and other neuroinfections, while remaining low in MS and other neuroinflammatory conditions [42]. Pyroglutamate, which converts to glutamate before being incorporated into GSH and also activates amino acid transport systems at the blood brain barrier [43], was higher in histoplasmosis, Lyme disease and WNV and was an important predictor distinguishing these conditions from control samples. Given individual metabolites can participate in a number of biochemical pathways, further studies are required to parse out the mechanisms at play in the diseases studied here. A likely interpretation is that infection or inflammation in the CNS is associated with redox imbalances including glutathione metabolism and NADH/NAD^+^ ratios. It is of particular interest that these metabolites may profile mechanisms leading to insulin resistance and vascular disease [36], given that low dose insulin therapy was added to the Milwaukee protocol, version 4, with statistical improvements in survival [44].

Our analytical design sought to minimize the effects of starvation/ketosis and dehydration/uremia on the metabolic profile of rabies by prioritizing rabies samples taken four days after admission. Nevertheless, PCA analysis identified the importance of ketone bodies in identifying rabies. Factor analysis that deliberately excluded primary ketones, urea and creatinine from analysis still identified isopropanol and methanol (Table 3), both downstream metabolites of ketones, as discriminators of rabies. RF and CART analyses also identified ketones and carnitine (fatty acid oxidation) as predictors of rabies but not other infections (Table 5). Despite our experimental design, CNS ketosis may be a valid indicator of rabies encephalitis.

This study was originally intended to further explore the specificity of NMR metabolomics for the diagnosis of rabies, which is often confused with Guillain-Barre syndrome, acute psychosis and N-methyl-D-aspartate receptor (NMDAR) encephalitis and currently requires multiple tests for diagnosis at remote reference laboratories. Our findings suggest that the utility of the approach may instead lie in excluding competing diagnoses, many of which are more treatable. NMR metabolomics performed on a par with current rabies diagnostics (100% sensitivity, 76% specificity) and is likely complementary (particularly after 5 days). When restricted to the first week of hospitalization with rabies (when most patients die), NMR metabolomics did not perform as well as for other infections; gene expression studies of rabies CSF and detection of rabies-specific antibodies also performed poorly in the first week. Rabies can clearly be delineated from controls by NMR at later time points, and NMR of CSF also measures recovery [12]. The promise of an NMR metabolomics profile as a proxy marker for therapeutic response would be welcome for rabies, WNV, NMDAR encephalitis or acute disseminated encephalomyelitis for which efficacious treatments remain undefined.

This study is exploratory and is limited by the number of samples available for CNS diseases of rare incidence. The possibility of confounding effects of age, sex, disease stage, or other acute variations in metabolic processes should be considered in interpreting these results. Our control group was aged 5–20 years, while ages in the disease group ranged from 4 to 83 years. However, we confirmed that the distribution of metabolites of our controls overlapped with adult norms reported by the international Human Metabolomics Database (www.hmdb.ca). Further, clear inter-disease differences within groups of adult diseases (MS, WNV) were evident in PCA (Fig 2), suggesting disease was much more influential in driving variation than was age. Sensitivity analyses in rabies in a larger dataset [12] did not identify meaningful age differences, although we cannot exclude the possibility that this might occur for other inflammatory diseases of the CNS. Another potential source of confounding is the timing of sample collection, which was not precisely known for samples other than rabies. All forms of encephalitis are treated empirically upon hospitalization, so early diagnostic samples such as those analyzed here may reflect early empirical therapies that often overlap (e.g., rehydration, provision of glucose, use of antibacterials, sedation) but may also differ between diseases. Our choice of rabies samples centered on the fourth day of hospitalization was intended to minimize effects of dehydration and malnutrition, but may have biased rabies samples toward normality. Finally, differences in some metabolites should be interpreted with caution, since low concentrations in some specimens precluded exact quantification (carnitine and glycine), which may have artificially led to statistical differences. Other metabolites (glutamine and pyroglutamate) are potentially affected by protein removal [45], although this has not been shown in CSF.

This study provides justification for further analysis of samples from these and other causes of encephalomyelitis. Several prominent and as of yet unidentified peaks observed in the spectra of some patients may indicate the presence of important metabolites involved in disease pathogenesis that have not yet been elucidated. While further studies with larger sample sizes will be needed to determine the clinical utility of NMR in the diagnosis of EM, NMR or other ‘omics technologies may in the future serve as a rapid initial screening test that would allow medical practitioners to initiate treatment with antivirals or biological immune modifiers, while patient samples can then be triaged to appropriate reference laboratories for confirmation without delaying treatment. Rabies and many arbovirus reference laboratories require specialized containment facilities, immunization of laboratory workers, and highly trained personnel who perform subjective assays such as immunofluorescence. Reference laboratories for rabies, arboviruses, bacteria and fungi are often dispersed geographically, leading to substantial requirements in volume, delay, and cost for diagnosis of encephalomyelitis when all are considered. NMR and MS instruments, on the other hand, exist at most research universities, i.e. at a state or provincial rather than national level. NMR analytical procedures are easily standardized and permit detection of multiple diseases using a single experiment, as illustrated here. NMR spectra can be transmitted electronically for analysis, which can be automated [46]. Decision analytical approaches such as CART and RF offer diagnostic flow charts that are easily implemented once validated, with quantifiable diagnostic probabilities. Considering current challenges, its relative ease of use makes NMR metabolomics of CSF a potentially important tool for emergent diseases and distinguishing between autoimmune and infectious EM.

## Supporting information

S1 Table Concentrations (μM) of metabolites in cerebrospinal fluid by infection status measured by 1H NMR spectroscopy. (PDF)

S2 Table Concentrations (μM) of metabolites in cerebrospinal fluid by diagnosis. (PDF)

S1 Dataset Metabolite concentration data. (CSV)

## References

[1] Britton PN, Eastwood K, Paterson B, Durrheim DN, Dale RC, Cheng AC, et al Consensus guidelines for the investigation and management of encephalitis in adults and children in Australia and New Zealand. Intern Med J. 2015;45(5):563–76. 10.1111/imj.12749 25955462

[2] Venkatesan A, Tunkel AR, Bloch KC, Lauring AS, Sejvar J, Bitnun A, et al Case definitions, diagnostic algorithms, and priorities in encephalitis: consensus statement of the international encephalitis consortium. Clin Infect Dis. 2013;57(8):1114–28. 10.1093/cid/cit458 23861361 PMC3783060

[3] Kneen R, Michael BD, Menson E, Mehta B, Easton A, Hemingway C, et al Management of suspected viral encephalitis in children—Association of British Neurologists and British Paediatric Allergy, Immunology and Infection Group national guidelines. J Infect. 2012;64(5):449–77. 10.1016/j.jinf.2011.11.013 22120594

[4] Sharma S, Mishra D, Aneja S, Kumar R, Jain A, Vashishtha VM, et al Consensus guidelines on evaluation and management of suspected acute viral encephalitis in children in India. Indian Pediatr. 2012;49(11):897–910. 23255700 10.1007/s13312-012-0216-0

[5] Granerod J, Ambrose HE, Davies NW, Clewley JP, Walsh AL, Morgan D, et al Causes of encephalitis and differences in their clinical presentations in England: a multicentre, population-based prospective study. Lancet Infect Dis. 2010;10(12):835–44. 10.1016/S1473-3099(10)70222-X 20952256

[6] Scharton D, Bailey KW, Vest Z, Westover JB, Kumaki Y, Van Wettere A, et al Favipiravir (T-705) protects against peracute Rift Valley fever virus infection and reduces delayed-onset neurologic disease observed with ribavirin treatment. Antiviral Res. 2014;104:84–92. 10.1016/j.antiviral.2014.01.016 24486952 PMC3975078

[7] Pohl D, Tenembaum S. Treatment of acute disseminated encephalomyelitis. Curr Treat Options Neurol. 2012;14(3):264–75. 10.1007/s11940-012-0170-0 22476745

[8] Kahn I, Helman G, Vanderver A, Wells E. Anti- N-Methyl-d-Aspartate (NMDA) Receptor Encephalitis. J Child Neurol. 2017;32(2):243–5. 10.1177/0883073816675557 27872179

[9] Smolinska A, Blanchet L, Buydens LM, Wijmenga SS. NMR and pattern recognition methods in metabolomics: from data acquisition to biomarker discovery: a review. Analytica chimica acta. 2012;750:82–97. 10.1016/j.aca.2012.05.049 23062430

[10] Rolland T, Tasan M, Charloteaux B, Pevzner SJ, Zhong Q, Sahni N, et al A proteome-scale map of the human interactome network. Cell. 2014;159(5):1212–26. 10.1016/j.cell.2014.10.050 25416956 PMC4266588

[11] Villoslada P, Baranzini S. Data integration and systems biology approaches for biomarker discovery: challenges and opportunities for multiple sclerosis. Journal of neuroimmunology. 2012;248(1–2):58–65. 10.1016/j.jneuroim.2012.01.001 22281286

[12] O'Sullivan A, Willoughby RE, Mishchuk D, Alcarraz B, Cabezas-Sanchez C, Condori RE, et al Metabolomics of cerebrospinal fluid from humans treated for rabies. Journal of proteome research. 2013;12(1):481–90. 10.1021/pr3009176 23163834 PMC4824192

[13] Pieragostino D, D'Alessandro M, di Ioia M, Rossi C, Zucchelli M, Urbani A, et al An integrated metabolomics approach for the research of new cerebrospinal fluid biomarkers of multiple sclerosis. Molecular bioSystems. 2015. 10.1039/c4mb00700j 25690641

[14] Coen M, O'Sullivan M, Bubb WA, Kuchel PW, Sorrell T. Proton nuclear magnetic resonance-based metabonomics for rapid diagnosis of meningitis and ventriculitis. Clin Infect Dis. 2005;41(11):1582–90. 10.1086/497836 16267730

[15] Subramanian A, Gupta A, Saxena S, Gupta A, Kumar R, Nigam A, et al Proton MR CSF analysis and a new software as predictors for the differentiation of meningitis in children. NMR in biomedicine. 2005;18(4):213–25. 10.1002/nbm.944 15627241

[16] Molins CR, Ashton LV, Wormser GP, Hess AM, Delorey MJ, Mahapatra S, et al Development of a metabolic biosignature for detection of early Lyme disease. Clin Infect Dis. 2015;60(12):1767–75. 10.1093/cid/civ185 25761869 PMC4810808

[17] Molins CR, Ashton LV, Wormser GP, Andre BG, Hess AM, Delorey MJ, et al Metabolic differentiation of early Lyme disease from southern tick-associated rash illness (STARI). Sci Transl Med. 2017;9(403). 10.1126/scitranslmed.aal2717 PMC5773101 28814545

[18] Wishart DS, Lewis MJ, Morrissey JA, Flegel MD, Jeroncic K, Xiong Y, et al The human cerebrospinal fluid metabolome. Journal of chromatography B, Analytical technologies in the biomedical and life sciences. 2008;871(2):164–73. 10.1016/j.jchromb.2008.05.001 18502700

[19] Reinke SN, Broadhurst DL, Sykes BD, Baker GB, Catz I, Warren KG, et al Metabolomic profiling in multiple sclerosis: insights into biomarkers and pathogenesis. Multiple sclerosis. 2014;20(10):1396–400. 10.1177/1352458513516528 24468817

[20] Lutz NW, Viola A, Malikova I, Confort-Gouny S, Audoin B, Ranjeva JP, et al Inflammatory multiple-sclerosis plaques generate characteristic metabolic profiles in cerebrospinal fluid. PloS one. 2007;2(7):e595 10.1371/journal.pone.0000595 17611627 PMC1899231

[21] Kork F, Holthues J, Hellweg R, Jankowski V, Tepel M, Ohring R, et al A possible new diagnostic biomarker in early diagnosis of Alzheimer's disease. Current Alzheimer research. 2009;6(6):519–24. 19747162 10.2174/156720509790147160

[22] Borroni B, Premi E, Di Luca M, Padovani A. Combined biomarkers for early Alzheimer disease diagnosis. Curr Med Chem 2007;14(11):1171–8. 17504137 10.2174/092986707780598005

[23] Ohman A, Forsgren L. NMR metabonomics of cerebrospinal fluid distinguishes between Parkinson's disease and controls. Neuroscience letters. 2015;594:36–9. 10.1016/j.neulet.2015.03.051 25817365

[24] Nicoli F, Vion-Dury J, Maloteaux JM, Delwaide C, Confort-Gouny S, Sciaky M, et al CSF and serum metabolic profile of patients with Huntington's chorea: a study by high resolution proton NMR spectroscopy and HPLC. Neuroscience letters. 1993;154(1–2):47–51. 8361646 10.1016/0304-3940(93)90168-k

[25] Noga MJ, Dane A, Shi S, Attali A, van Aken H, Suidgeest E, et al Metabolomics of cerebrospinal fluid reveals changes in the central nervous system metabolism in a rat model of multiple sclerosis. Metabolomics: Official journal of the Metabolomic Society. 2012;8(2):253–63. 22448154 10.1007/s11306-011-0306-3 PMC3291832

[26] Bacon RM, Kugeler KJ, Mead PS, Centers for Disease C, Prevention. Surveillance for Lyme disease—United States, 1992–2006. Morbidity and mortality weekly report Surveillance summaries. 2008;57(10):1–9. 18830214

[27] Reimann CA, Hayes EB, DiGuiseppi C, Hoffman R, Lehman JA, Lindsey NP, et al Epidemiology of neuroinvasive arboviral disease in the United States, 1999–2007. The American journal of tropical medicine and hygiene. 2008;79(6):974–9. 19052314

[28] Mallewa M, Fooks AR, Banda D, Chikungwa P, Mankhambo L, Molyneux E, et al Rabies encephalitis in malaria-endemic area, Malawi, Africa. Emerg Infect Dis. 2007;13(1):136–9. 10.3201/eid1301.060810 17370529 PMC2725806

[29] Carrithers MD. Innate immune viral recognition: relevance to CNS infections. Handbook of clinical neurology. 2014;123:215–23. 10.1016/B978-0-444-53488-0.00009-2 25015487

[30] Klein RS, Hunter CA. Protective and Pathological Immunity during Central Nervous System Infections. Immunity. 2017;46(6):891–909. 10.1016/j.immuni.2017.06.012 28636958 PMC5662000

[31] Luckhart S, Pakpour N, Giulivi C. Host-pathogen interactions in malaria: cross-kingdom signaling and mitochondrial regulation. Current opinion in immunology. 2015;36:73–9. 10.1016/j.coi.2015.07.002 26210301 PMC4593738

[32] Cliff N. Answering Ordinal Questions with Ordinal Data Using Ordinal Statistics. Multivariate Behav Res. 1996;31(3):331–50. 10.1207/s15327906mbr3103_4 26741071

[33] Henrard S, Speybroeck N, Hermans C. Classification and regression tree analysis vs. multivariable linear and logistic regression methods as statistical tools for studying haemophilia. Haemophilia. 2015;21(6):715–22. 10.1111/hae.12778 26248714

[34] Touw WG, Bayjanov JR, Overmars L, Backus L, Boekhorst J, Wels M, et al Data mining in the Life Sciences with Random Forest: a walk in the park or lost in the jungle? Brief Bioinform. 2013;14(3):315–26. 10.1093/bib/bbs034 22786785 PMC3659301

[35] Landaas S. The formation of 2-hydroxybutyric acid in experimental animals. Clin Chim Acta. 1975;58(1):23–32. 164303 10.1016/0009-8981(75)90481-7

[36] Gall WE, Beebe K, Lawton KA, Adam KP, Mitchell MW, Nakhle PJ, et al alpha-hydroxybutyrate is an early biomarker of insulin resistance and glucose intolerance in a nondiabetic population. PloS one. 2010;5(5):e10883 10.1371/journal.pone.0010883 20526369 PMC2878333

[37] Cook JS, Pogson CI. Effect of 4-hydroxypyrazole on tryptophan and formate metabolism in isolated rat liver cells. Biochem J. 1982;204(1):307–12. 6810879 10.1042/bj2040307 PMC1158346

[38] Thony B, Auerbach G, Blau N. Tetrahydrobiopterin biosynthesis, regeneration and functions. Biochem J. 2000;347 Pt 1:1–16. 10727395 PMC1220924

[39] Lee ES, Chen H, Hardman C, Simm A, Charlton C. Excessive S-adenosyl-L-methionine-dependent methylation increases levels of methanol, formaldehyde and formic acid in rat brain striatal homogenates: possible role in S-adenosyl-L-methionine-induced Parkinson's disease-like disorders. Life Sci. 2008;83(25–26):821–7. 10.1016/j.lfs.2008.09.020 18930743 PMC2885904

[40] Lamarre SG, Molloy AM, Reinke SN, Sykes BD, Brosnan ME, Brosnan JT. Formate can differentiate between hyperhomocysteinemia due to impaired remethylation and impaired transsulfuration. Am J Physiol Endocrinol Metab. 2012;302(1):E61–7. 10.1152/ajpendo.00345.2011 21934042 PMC3328090

[41] Willoughby RE, Opladen T, Maier T, Rhead W, Schmiedel S, Hoyer J, et al Tetrahydrobiopterin deficiency in human rabies. J Inherit Metab Dis. 2009;32(1):65–72. 10.1007/s10545-008-0949-z 18949578

[42] Hytonen J, Kortela E, Waris M, Puustinen J, Salo J, Oksi J. CXCL13 and neopterin concentrations in cerebrospinal fluid of patients with Lyme neuroborreliosis and other diseases that cause neuroinflammation. J Neuroinflammation. 2014;11:103 10.1186/1742-2094-11-103 24920219 PMC4070086

[43] Hawkins RA, O'Kane RL, Simpson IA, Vina JR. Structure of the blood-brain barrier and its role in the transport of amino acids. The Journal of nutrition. 2006;136(1 Suppl):218S–26S. 10.1093/jn/136.1.218S 16365086

[44] Willoughby RE, Jr. Rabies treatment protocol and registry. www.mcw.edu/rabies 2018(5). Available from: URL: www.mcw.edu/rabies

[45] Nagana Gowda GA, Gowda YN, Raftery D. Massive glutamine cyclization to pyroglutamic acid in human serum discovered using NMR spectroscopy. Anal Chem. 2015;87(7):3800–5. 10.1021/ac504435b 25746059 PMC4479397

[46] Ravanbakhsh S, Liu P, Bjordahl TC, Mandal R, Grant JR, Wilson M, Eisner R, Sinelnikov I, Hu X, Luchinat C, Greiner R. Accurate, fully-automated NMR spectral profiling for metabolomics. PLoS One. 2015 5 27;10(5):e0124219 10.1371/journal.pone.0124219 26017271 PMC4446368

