# Supplementary material for: NMR metabolomics of cerebrospinal fluid differentiates inflammatory diseases of the central nervous system
Source: PLoS Negl Trop Dis. 2018 Dec 17;12(12):e0007045. doi: 10.1371/journal.pntd.0007045 (PMC6312347; doi:10.1371/journal.pntd.0007045)
Supplement: S1 Table — (PDF) [file pntd.0007045.s001.pdf]

S1 Table. Concentrations (μM) of metabolites in cerebrospinal fluid by infection status measured by ^{1}H NMR spectroscopy

	No Infection		Infection	
**Metabolite**	Median	(IQR)	Median	(IQR)
2-Hydroxybutyrate	33.89	(12.48)	59.78	(64.67)
2-Hydroxyisovalerate	5.34	(3.20)	7.27	(5.83)
2-Oxoglutarate	1.78	(2.33)[a]	2.89	(2.61)[a]
2-Oxoisocaproate	1.22	(0.56)[a]	1.78	(1.90)[a]
3-Hydroxybutyrate	7.22	(6.49)	48.78	(185.72)
3-Hydroxyisobutyrate	13.67	(4.14)	18.44	(9.46)
3-Hydroxyisovalerate	2.65	(1.83)	3.89	(3.09)
Acetamide	2.33	(1.22)[a]	2.00	(2.83)[a]
Acetate	106.65	(35.49)	59.78	(66.81)
Acetoacetate	6.22	(2.98)	22.44	(71.39)
Acetone	4.18	(9.99)	24.67	(80.56)
Alanine	26.33	(10.67)	31.33	(28.18)
Betaine	2.44	(0.78)[a]	3.56	(1.60)[a]
Carnitine	1.69	(0.97)[a]	3.44	(2.22)[a]
Choline	1.89	(0.76)	2.67	(2.66)
Citrate	172.11	(40.59)	185.33	(106.71)
Creatine	45.19	(8.69)	49.11	(18.23)
Creatinine	51.56	(14.66)	49.78	(21.71)
Dimethyl sulfone	3.80	(2.61)	3.22	(3.51)
Ethanolamine	16.11	(5.46)	14.49	(10.67)
Formate	26.86	(9.78)	45.22	(27.69)
Fructose	124.67	(53.13)	80.44	(41.67)
Fucose	3.89	(2.19)[a]	3.78	(2.49)[a]
Glucose	3294.73	(722.07)	2993.24	(1787.33)
Glutamate	2.82	(3.01)[a]	8.48	(8.33)[a]
Glutamine	443.99	(99.01)	389.78	(283.67)
Glycine	4.88	(2.53)[a]	13.71	(15.99)
Glycolate	8.22	(2.19)	8.44	(3.67)

S1 Table (continued)

	No Infection		Infection	
**Metabolite**	Median	(IQR)	Median	(IQR)
Isobutyrate	1.27	(1.67)	0.79	(0.67)
Isoleucine	4.51	(1.38)	5.44	(3.75)
Isopropanol	2.00	(1.42)	9.56	(8.00)
Lactate	1542.75	(316.08)	1814.11	(1019.14)
Leucine	10.95	(3.45)	13.78	(9.85)
Lysine	20.00	(8.67)	19.22	(8.16)
Methanol[b]	19.96	(11.52)	14.33	(9.06)
Methionine	3.02	(1.27)[a]	3.11	(3.46)[a]
N-acetylaspartate	0.93	(0.56)[a]	1.44	(1.06)[a]
N-acetylneuraminate	16.33	(7.33)	11.50	(6.18)
NAAG	1.55	(1.33)	1.08	(1.20)
Ornithine	3.31	(1.89)[a]	4.56	(4.60)[a]
Phenylalanine	8.44	(4.75)	13.16	(8.43)
Propionate	1.93	(1.38)[a]	1.89	(1.48)[a]
Pyroglutamate	19.44	(7.34)	61.33	(81.25)
Pyruvate	28.05	(44.13)	77.78	(81.50)
Quinolinate	1.08	(1.63)[a]	3.09	(3.17)[a]
Serine	33.43	(11.83)	23.56	(11.27)
Threonine	31.96	(16.22)	31.11	(21.14)
Tyrosine	10.91	(4.86)	10.70	(7.55)
Urea	1947.89	(1220.18)	2719.23	(2441.61)
Valine	15.41	(5.59)	19.00	(13.86)
myo-Inositol	118.78	52.67	99.51	(39.93)

Abbreviations: IQR, interquartile range

[a]Median concentrations are not accurate due to concentrations for many samples being below the detection limit.

[b]Tentatively assigned due to having only one quantifiable peak and unexplained chemical shift in the NMR spectra.
