# Supplementary material for: NMR metabolomics of cerebrospinal fluid differentiates inflammatory diseases of the central nervous system
Source: PLoS Negl Trop Dis. 2018 Dec 17;12(12):e0007045. doi: 10.1371/journal.pntd.0007045 (PMC6312347; doi:10.1371/journal.pntd.0007045)
Supplement: S2 Table — (PDF) [file pntd.0007045.s002.pdf]

S2 Table. Concentrations (μM) of metabolites in cerebrospinal fluid by diagnosis

	Control		Multiple Sclerosis		West Nile Virus		Lyme		Rabies		Fungal	
**Metabolite**	Median	(IQR)	Median	(IQR)	Median	(IQR)	Median	(IQR)	Median	(IQR)	Median	(IQR)
2-Hydroxybutyrate	32.68	(13.02)	39.11	(13.83)	133.67	(85.11)	42.56	(25.72)	62.06	(44.56)	24.33	(11.94)
2-Hydroxyisovalerate	5.00	(3.20)	5.67	(2.25)	10.56	(0.33)	4.78	(1.66)	9.03	(9.35)	4.67	(0.83)
2-Oxoglutarate[a]	1.78	(2.33)	1.11	(0.56)	4.89	(4.44)	3.67	(3.33)	2.40	(1.31)	1.44	(0.00)
2-Oxoisocaproate[a]	1.25	(0.66)	1.17	(0.31)	2.67	(2.44)	1.78	(0.78)	1.81	(1.67)	1.11	(0.44)
3-Hydroxybutyrate	7.41	(11.29)	7.17	(0.67)	259.00	(312.89)	48.78	(36.00)	68.17	(197.77)	15.44	(19.00)
3-Hydroxyisobutyrate	13.67	(4.26)	13.72	(1.81)	22.33	(6.56)	10.67	(5.82)	22.74	(6.33)	17.67	(3.83)
3-Hydroxyisovalerate	2.65	(1.83)	2.94	(1.86)	6.44	(3.00)	2.33	(0.42)	4.32	(3.06)	4.00	(0.67)
Acetamide[a]	2.17	(1.29)	2.44	(0.50)	2.89	(3.00)	4.11	(0.93)	1.32	(1.19)	5.00	(3.22)
Acetate	106.65	(27.13)	96.72	(107.19)	33.78	(10.22)	59.78	(28.89)	83.39	(66.37)	85.33	(91.61)
Acetoacetate	6.39	(4.88)	5.61	(1.33)	75.33	(106.11)	26.22	(11.03)	24.63	(92.11)	1.89	(3.89)
Acetone	4.75	(10.47)	3.61	(0.97)	98.89	(39.11)	25.56	(14.57)	22.90	(164.52)	7.22	(3.28)
Alanine	25.10	(9.63)	31.44	(4.50)	40.67	(29.44)	29.11	(3.89)	29.89	(26.54)	35.00	(17.39)
Betaine[a]	2.45	(0.72)	2.22	(0.50)	5.11	(2.56)	3.44	(0.33)	2.90	(2.33)	3.56	(0.17)
Carnitine[a]	1.67	(0.75)	2.50	(0.53)	4.22	(0.33)	2.78	(1.11)	1.62	(2.06)	3.44	(2.72)
Choline	1.89	(0.84)	2.00	(0.75)	4.22	(1.78)	3.00	(0.56)	2.10	(1.77)	5.78	(2.33)
Citrate	169.44	(40.88)	190.17	(50.86)	178.67	(99.22)	185.33	(124.34)	180.87	(77.58)	252.78	(44.22)
Creatine	45.19	(11.69)	46.06	(4.50)	49.56	(15.67)	47.00	(15.44)	42.63	(10.74)	62.00	(9.06)
Creatinine	51.56	(12.85)	56.17	(17.56)	48.11	(32.11)	50.22	(4.11)	47.90	(24.38)	79.67	(40.94)

S2 Table (continued)

	Control		Multiple Sclerosis		West Nile Virus		Lyme		Rabies		Fungal	
**Metabolite**	Median	(IQR)	Median	(IQR)	Median	(IQR)	Median	(IQR)	Median	(IQR)	Median	(IQR)
Dimethyl sulfone	3.67	(2.44)	7.94	(1.42)	5.67	(2.00)	3.89	(1.22)	1.54	(1.61)	7.56	(3.44)
Ethanolamine	16.13	(5.44)	12.22	(4.97)	20.22	(12.89)	15.89	(8.67)	10.47	(12.24)	14.67	(0.67)
Formate	25.76	(8.80)	40.33	(21.39)	73.11	(31.00)	61.33	(23.56)	31.80	(3.38)	45.22	(2.17)
Fructose	125.55	(60.64)	118.11	(18.56)	87.11	(4.67)	58.78	(25.93)	85.94	(96.52)	79.67	(26.06)
Fucose[a]	3.99	(2.14)	2.06	(2.47)	4.33	(0.89)	1.84	(2.00)	3.97	(3.14)	3.22	(6.17)
Glucose	3345.44	(453.63)	2530.83	(211.92)	3240.56	(746.22)	2400.78	(380.10)	4128.06	(755.06)	2205.11	(400.61)
Glutamate[a]	3.01	(2.25)	0.89	(1.64)	15.67	(53.44)	11.78	(6.44)	6.29	(3.43)	8.67	(3.39)
Glutamine	445.56	(105.63)	414.72	(51.56)	240.00	(327.89)	288.11	(117.46)	519.33	(209.91)	261.67	(267.00)
Glycine	4.88	(2.27)[b]	5.94	(2.67)[a]	19.22	(8.56)	15.56	(8.00)	10.13	(21.79)	6.22	(0.89)
Glycolate	8.67	(2.19)	7.67	(1.33)	8.44	(1.00)	8.22	(1.56)	6.70	(3.55)	10.78	(7.22)
Isobutyrate	1.27	(0.86)	1.83	(1.83)	1.22	(0.33)	0.67	(0.22)	0.78	(0.43)	1.56	(0.94)
Isoleucine	4.33	(1.28)	5.33	(1.00)	6.33	(5.67)	5.44	(1.89)	5.15	(4.26)	4.33	(2.22)
Isopropanol	1.81	(1.41)	2.50	(1.33)	4.89	(7.00)	13.11	(3.78)	9.87	(6.58)	4.33	(2.89)
Lactate	1542.75	(316.08)	1554.33	(208.31)	2717.11	(1690.56)	1716.44	(125.78)	2072.21	(1142.34)	1511.11	(185.83)
Leucine	10.95	(4.20)	11.06	(2.72)	14.11	(16.22)	13.78	(2.78)	13.44	(10.16)	11.00	(2.67)
Lysine	19.85	(8.91)	20.78	(2.22)	19.22	(2.33)	15.44	(4.38)	19.85	(11.95)	22.56	(7.22)
Methanol[c]	20.78	(10.07)	12.00	(12.89)	9.56	(8.78)	14.44	(0.44)	16.71	(12.54)	13.44	(3.28)
Methionine[a]	3.10	(1.21)	2.56	(1.22)	2.67	(3.78)	1.89	(0.89)	4.23	(5.21)	3.11	(0.72)

S2 Table (continued)

	Control		Multiple Sclerosis		West Nile Virus		Lyme		Rabies		Fungal	
**Metabolite**	Median	(IQR)	Median	(IQR)	Median	(IQR)	Median	(IQR)	Median	(IQR)	Median	(IQR)
N-acetylaspartate[a]	0.93	(0.60)	0.89	(0.31)	0.89	(1.00)	1.44	(0.33)	1.39	(1.44)	1.67	(0.50)
N-acetylneuraminate	17.89	(6.50)	13.78	(1.97)	10.89	(2.00)	11.50	(6.56)	13.33	(3.68)	22.44	(12.83)
NAAG	2.05	(1.00)	0.72	(0.19)	2.11	(0.89)	1.15	(1.00)	0.97	(0.52)	1.00	(0.50)
Ornithine	3.71	(1.85)[b]	1.89	(2.61)[a]	2.44	(2.22)[b]	4.56	(0.89)[b]	4.69	(3.69)[b]	9.11	(2.33)
Phenylalanine	9.56	(4.82)	7.17	(1.72)	17.22	(6.11)	8.56	(4.22)	14.60	(4.99)	9.11	(10.39)
Propionate[a]	1.67	(0.74)	6.94	(8.50)	2.00	(0.44)	2.89	(2.00)	1.67	(1.33)	1.89	(0.39)
Pyroglutamate	20.40	(7.35)	16.39	(3.25)	89.44	(32.56)	65.00	(118.00)	31.15	(21.57)	146.78	(192.06)
Pyruvate	28.05	(41.51)	39.83	(47.75)	195.11	(48.11)	54.67	(18.04)	80.73	(75.16)	9.44	(17.17)
Quinolinate[a]	1.03	(1.23)	1.89	(1.14)	4.67	(2.67)	3.44	(2.11)	1.80	(3.60)	3.44	(1.67)
Serine	32.84	(11.50)	37.67	(10.17)	23.56	(6.56)	27.33	(8.19)	22.00	(18.29)	17.11	(3.22)
Threonine	35.51	(19.35)	27.72	(4.64)	33.67	(36.67)	27.89	(7.33)	32.93	(21.22)	30.22	(11.83)
Tyrosine	11.84	(4.14)	9.56	(2.25)	10.11	(3.89)	6.56	(1.22)	11.93	(8.47)	8.11	(18.56)
Urea	1893.19	(997.62)	2547.61	(802.39)	2129.44	(3278.89)	2397.11	(506.22)	3025.47	(2987.82)	4554.56	(5698.22)
Valine	15.41	(5.59)	16.00	(4.78)	28.56	(22.56)	19.00	(9.33)	16.62	(13.76)	19.56	(4.72)
myo-Inositol	118.78	(54.40)	103.11	(50.64)	86.44	(36.56)	97.33	(31.67)	106.49	(38.70)	137.89	(52.11)

Abbreviations: IQR, interquartile range

[a]Median concentrations are not accurate due to concentrations for many samples being below the detection limit.

[b]Tentatively assigned due to having only one quantifiable peak and unexplained chemical shift in the NMR spectra.
